# A regional comparative study on the mismatch between population urbanization and land urbanization in China

**DOI:** 10.1371/journal.pone.0287366

**Published:** 2023-06-30

**Authors:** Xingfen Wang, Xindi Zhang

**Affiliations:** School of Marxism, Yunnan Normal University, Kunming, Yunnan, le; Sam Houston State University, UNITED STATES

## Abstract

By taking 31 provinces (municipalities/autonomous regions) in Mainland China as the object of research, and using the data on urban population and built-up area of each region from 2005 to 2019, this paper measures the dispersion coefficient of population urbanization and land urbanization in each region through models and visually expresses the level and type of imbalance between them to reveal the temporal and spatial characteristics of imbalance. The results of the research show that since China’s state-owned land was sold through bidding, auction, and listing, the overall urbanization of the population and land development have become unbalanced. There is obvious regional and category difference in imbalance between population urbanization and land urbanization. The degree of imbalance increases from the central, eastern, northeastern to western regions. The remaining 29 regions are generally lagging in population urbanization except for Beijing and Hebei province. This imbalance is mainly caused by China’s dual household registration system, dual land system and the asymmetrical tax distribution system between financial rights and administrative rights.

## 1. Introduction

**Fig 1:**
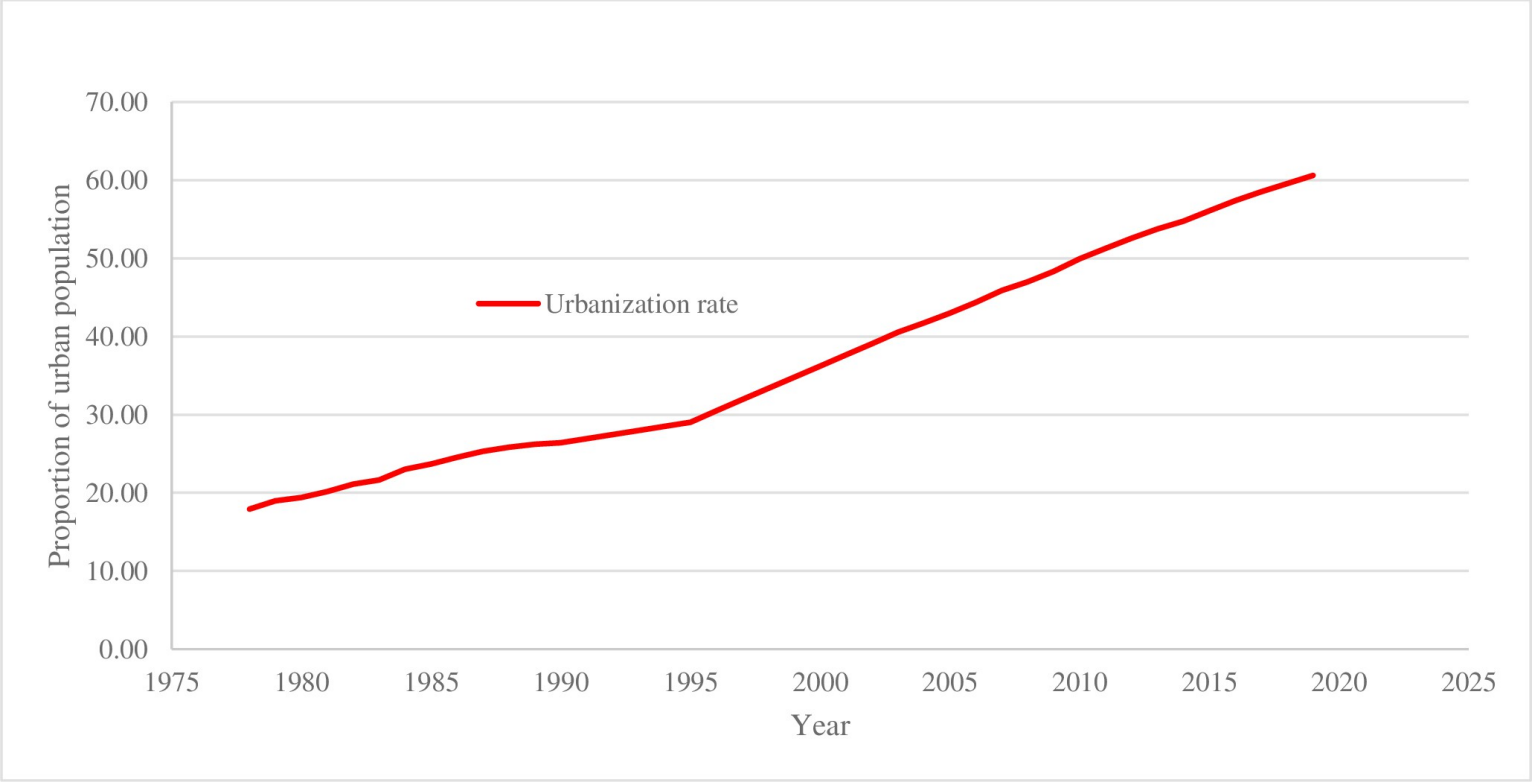
Urbanization rate from 1978 to 2019 in China. Source: National Bureau of Statistics: stats.gov.cn.

Since the implementation of reform and opening up policy, China has sustained rapid economic growth and economic globalization which have led to rapid development of urbanization. From 1978 to 2019, the proportion of China’s urban population rose from 17.92% to 60.6% (Fig 1). Over the past 42 years, urbanization has increased by an average annual rate of 1.04 percentage points. According to the general law of urbanization development, China’s urbanization is still in the stage of rapid development. In many countries, this stage is the golden period of rapid economic development as well as a period full of frequent problems [1]. Urbanization is an important starting point for accelerating the process of upgrading industrial structures and is a powerful engine for leveraging domestic demands and maintaining sustained and healthy economic development [2]. Therefore, whether urbanization can achieve comprehensive, coordinated, and sustainable development plays a key role in China’s economic development and has a profound impact on the living conditions of Chinese residents [3].

The 19th National Congress of the Communist Party of China in 2017 proposed a rural revitalization strategy. The central No. 1 document of 2018 comprehensively explained the significance and overall requirements of the rural revitalization strategy, as well as clarified its development thinking of the rural revitalization strategy. The central No. 1 document of 2019 and 2020 both focused on the three issues with agriculture, rural areas, and farmers. The status and role of rural areas were highlighted, and future urbanization was shown to need intensive development. While fully affirming the status of the countryside and vigorously promoting rural construction, how will the key elements of people and land be allocated? The coordinated development of population and land in the process of urbanization is an inevitable requirement for new urbanization.

The current Chinese research mainly focuses on the following points: In terms of research content, quantitative research on the evolution trend and coupling relationship of population urbanization and land urbanization was conducted involving the connotation of population urbanization and land urbanization [4–9], the degree measurement of population-land coordinated development [10–15], the analysis of influencing factors [6, 16–18], and optimization strategies [19–21]. In terms of research scale, existing studies just cover a certain region, province, city, district and county, etc. [22, 23]. In terms of research methods, in most existing studies, comprehensive index method was adopted, coupling coordination degree and other models to conduct quantitative research on man-land urbanization [24]. The main shortcoming of the existing research is that the index information of the comprehensive index method has errors and overlaps [25]. The difference between the existing research and this paper lies in the use of a single index method to compare the characteristics and differences of imbalance in different regions of China.

In view of this, the author took 31 provinces (municipalities/autonomous regions) in Mainland China as the sample, using the urban population and built-up area data of each province (municipalities/autonomous regions) from 2005 to 2019, in order to measure the dispersion coefficient of population urbanization and land urbanization in various provinces (municipalities/autonomous regions) through the model. The author also used ArcGIS software for visual expression to reveal the characteristics and differences of the imbalance between population urbanization and land urbanization in the process of rapid urbanization in China.

## 2. Relationship of population urbanization and land urbanization

“Population urbanization refers to the process of transformation of non-urban population (including farmers and migrant workers) into urban population, from working in the agricultural industry to working in the non-agricultural industry, in which their dual transformation of identity and occupation is realized, and equal citizen treatment obtained” [26].

“Land urbanization refers to the process of land conversion from rural land to urban construction land, and the expansion of spatial scale of cities and towns. In China, land urbanization not only refers to the ’agricultural conversion’ of land use, but also includes the transformation of the subject of land property rights from rural collectives to the country and the change in the nature of property rights from collective ownership to state ownership” [26].

Both population urbanization and land urbanization are important parts of urbanization. The former is the core, and “its essence is the spatial transfer process of human economic activities” [26]; the latter is the carrier, which is “mainly manifested in the increase of urban built-up areas” [12]. Urbanization can be defined as the coordinated development of them [27]. Healthy and sustainable urbanization is "people-oriented" and "land-based".

Population urbanization is the core. In the process of urbanization, people are always the most crucial factor. The starting point and landing point of an urbanization strategy must be reflected with people as the main component. This is because the emergence of urbanization originates from people, the development of urbanization depends on people, and the promotion of urbanization is for people.

Land urbanization is the carrier of space. As a necessary resource in the process of urbanization, land provides the space for the production and living areas of residents, including the land for residential housing, industrial production and commercial land, etc. Without land as a supporting condition, urbanization would be a "castle in the air". In addition, with the advancement of urbanization and the increase of urban population, the required space scale will also increase correspondingly.

Therefore, with the increase of urban population size, land urbanization should also be increased in proportion to maintain the normal production and living needs of the urban population. This requires the coordinated development of population urbanization and land urbanization. If land urbanization is faster than population urbanization, land resources will be wasted. However, if population urbanization is faster than land urbanization, urban population will be overcrowded. Only the coordinated development of population urbanization and land urbanization can result in healthy urbanization.

## 3. Methods and data

There are many methods to measure the coordination degree of population urbanization and land urbanization, but there are five more commonly used: dispersion coefficient method [10], coordination index method [28], coordinated development model [8, 9, 27, 29], ROXY method [30], and land growth elasticity coefficient [31, 32].

The coordination index is a quantitative index to measure the coordination among the elements of the system. The size of the coordination index can be calculated through the two indicators of urban population growth rate and the built-up area growth rate. The value range of the coordination index is [0,^1^]. When the coordination index is equal to 0, it means that population urbanization and land urbanization are the most inconsistent. When the coordination index is 1, it means that the two are the most harmonious. In short, the faster the matching speed of land urbanization and population urbanization, the higher the coordination index.

The coordinated development model is used to analyze the level of coordinated development of things, involving the calculation of three index values, namely, the degree of coordination, the degree of development and the degree of coordinated development. Combined with the value of the degree of coordinated development and the classification standard of coordination level, the coordinated development of population urbanization and land urbanization is judged and analyzed. The coordinated development model is one of the composite index methods, and the measured result is a relative number in the form of an exponential, which is comparable. At the same time, indicators that can be used to evaluate population urbanization and land urbanization can be selected as comprehensively as possible according to the connotation of population urbanization and land urbanization, so as to judge the degree of coordination or imbalance between the two more accurately. Therefore, this method is more suitable for analyzing the coordination between population urbanization and land urbanization in a country, but not applicable for analyzing the coordination between population urbanization and land urbanization among provinces (municipalities/autonomous regions), due to data and many indicators being difficult to find.

The ROXY index is based on the ratio of the weighted average to the arithmetic average of an urban population growth rate in order to study the spatial distribution of urban development, which can reflect the basic characteristics and behaviors of urban spatial cyclic movement. According to the ROXY index model, by calculating the ratio of weighted average to the arithmetic average of regional population growth, the characteristics of spatial circulation movement in the process of urban development and the flow tendency of population in core and peripheral areas can be described, so as to measure the stages from urbanization to urbanization, suburbanization, reverse urbanization and re-urbanization of urban agglomeration and economic belt. The purpose of the concentration and diffusion level of the spatial population is also shown.

The elasticity coefficient of urban land use growth—urban land use growth rate/urban population growth rate—is widely used in the world to measure the coordination relationship between population urbanization and land urbanization. Jan Gale proposed that the reasonable value of this coefficient is 1.12 [33]. This method has been recognized by many Chinese scholars. However, considering only the ratio relationship between urban land use growth rate and urban population growth rate, it is difficult to reflect the per capita index of a city. If the per capita built-up area of urban areas is still measured according to the elasticity coefficient of urban land growth of 1.12, it goes against the compact urban development and other land conservation concepts and cannot objectively reflect the coordination relationship between population urbanization and land urbanization.

The coefficient of deviation, also known as the coefficient of variation, is a relative quantity indicating the size of the standard deviation relative to the mean. The deviation coefficient is a relative number in the form of ratio, which can reflect the dispersion degree of different data sets [34] and clearly reflects the coordination state between population urbanization and land urbanization. The value range of the deviation coefficient is [0, +∞]. The coordination between population urbanization and land urbanization can be judged according to the size of the deviation coefficient. The smaller the deviation coefficient is, the more coordinated they are, and vice versa. Therefore, the coefficient of deviation method has been chosen for this study.

### 3.1. Deviation coefficient method

The coefficient of dispersion, also known as the coefficient of variation, is a relative quantity that represents the standard deviation relative to the average. The calculation formula of the dispersion coefficient is $C_{V}=\frac{S}{\left|\bar{X}\right|}$, with S being the standard deviation, $S=\sqrt{\frac{1}{n}\sum_{i=1}^{n}{\left(X_{i}-\bar{X}\right)}^{2}}$, and $\bar{X}$ being the average value $\bar{X}=\frac{\sum X_{i}}{n}$. Since this measurement only involves two systems of population urbanization and land urbanization, the calculation formula can therefore be derived as shown in Eq 1.


$$C_{V}=\frac{\sqrt{\frac{1}{2}[{\left(P-\frac{P+L}{2}\right)}^{2}+{\left(L-\frac{P+L}{2}\right)}^{2}]}}{\left|\frac{P+L}{2}\right|}=\left|\frac{P-L}{P+L}\right|$$ (1)

In Formula (1), C_V_ is the deviation coefficient, P is the growth rate of population in urban areas, and L is the growth rate of the built-up area.


$$\begin{array}{l} P=(\mathrm{urban}\;\mathrm{population}\;\mathrm{this}\;\mathrm{year}‐\mathrm{urban}\;\mathrm{population}\;\mathrm{last}\;\mathrm{year})/\mathrm{urban}\;\mathrm{population}\;\mathrm{last}\;\mathrm{year}\\ L=(\mathrm{built}‐\mathrm{up}\;\mathrm{area}\;\mathrm{this}\;\mathrm{year}‐\mathrm{built}‐\mathrm{up}\;\mathrm{area}\;\mathrm{last}\;\mathrm{year})/\mathrm{built}‐\mathrm{up}\;\mathrm{area}\;\mathrm{last}\;\mathrm{year} \end{array}$$ (2)

### 3.2. Index selection and data source

The dispersion coefficient method is a single index method. According to the connotation of population urbanization and land urbanization and the availability of data, the growth rate of the built-up area is selected to characterize land urbanization, and the urban population growth rate is used to characterize population urbanization. The data of urban population and the built-up area both are adopted from the China Statistical Yearbook.

In 2002, Ministry of Land and Resources Order No. 11 (the "Regulations on the Assignment of State-Owned Land Use Rights by Bidding, Auction and Listing") stipulated in Article 4 that "business, tourism, entertainment, commercial housing and other business land must be sold through bidding, auction or listing." In 2006, State Council Document No. 31 (the "Notice of the State Council on Relevant Issues Concerning Strengthening Land Regulation"), Article 5, stipulates that "Industrial land must be sold through bidding, auction and listing." That is to say, China’s state-owned land has been sold through bidding, auction, and listing since 2006. The author assumes that the rate of land urbanization has risen sharply since then. Therefore, the coordination of population urbanization and land urbanization in 31 provinces (municipalities/autonomous regions) of Mainland China from 2006 to 2019 is measured.

### 3.3. Assessment criteria

P stands for the urban population growth rate characterizing population urbanization, and L stands for the built-up area growth rate indicating land urbanization. When Max (P, L) = P, population urbanization is faster than land urbanization, suggesting a lagging type of land urbanization. When Max (P, L) = L, land urbanization is faster than population urbanization, showing a lagging type of population urbanization. When Max (P, L) = P = L, the dispersion coefficient is C_V_ = 0 at this time, it indicates the coordinated development of population urbanization and land urbanization.

The value range of the dispersion coefficient C_V_ is [0, +∞). The smaller the dispersion coefficient and the closer to zero, the more it indicates that the growth rate of population urbanization and land urbanization is equivalent, and the degree of out-of-control between population urbanization and land urbanization is smaller, and vice versa. The imbalance between population urbanization and land urbanization is divided into six levels (see Table 1). They are coordinated development when C_V_ ϵ [0.0, 0.2), mildly imbalanced when C_V_ ϵ [0.2, 0.4), moderately imbalanced when C_V_ ϵ [0.4, 0.6), highly imbalanced when C_V_ ϵ [0.6, 0.8), severely imbalanced when C_V_ ϵ [0.8, 1.0), extremely imbalanced when C_V_ ϵ [1.0, +∞), indicating that the imbalance between population urbanization and land urbanization is very serious (Yin and Xu, 2013).

**Table 1:** Classification criteria of imbalance between population urbanization and land urbanization.

Imbalance level	Extremely imbalanced	Severely imbalanced	Highly imbalanced	Moderately imbalanced	Mildly imbalanced	Coordinated development
C_V_	[1.0, +∞)	[0.8, 1.0)	[0.6, 0.8)	[0.4, 0.6)	[0.2, 0.4)	[0.0, 0.2)

**Source:** Yin H L, Xu T. The Mismatch between Population Urbanization and Land Urbanization in China. Urban Planning Forum. 2013;02: 10–15.

## 4. Results and discussions

### 4.1. Measurement results

First, using the data of urban population and the built-up area of 31 provinces (municipalities/autonomous regions) in Mainland China from 2005 to 2019 (S1 Appendix), the growth rate calculation Formula (2) is used to calculate the urban population growth rate P value (S2 Appendix) and the built-up area growth rate L value (S3 Appendix).

Second, the calculation Formula (1) of the dispersion coefficient method is used to calculate the dispersion coefficient C_V_ that reflects coordination (Tables 2 and 3).

**Table 2:** Annual C_V_ values of 31 provinces (municipalities/autonomous regions) in Mainland China.

Years	2006	2007	2008	2009	2010	2011	2012	2013	2014	2015	2016	2017	2018	2019
Beijing	0.05	0.27	0.57	0.27	3.41	0.08	0.02	0.22	0.54	0.03	0.79	1.13	3.03	1.00
Tianjin	0.35	0.13	0.29	0.25	0.35	0.22	0.57	0.16	0.33	0.64	0.83	1.08	2.96	0.85
Hebei	0.37	0.06	0.13	0.22	0.20	0.12	0.02	0.11	0.11	0.13	0.12	0.11	0.21	0.48
Shanghai	0.17	0.25	1.00	1.00	0.44	1.00	1.00	1.00	1.00	1.00	1.00	1.00	0.94	0.95
Jiangsu	0.40	0.19	0.46	0.23	0.14	0.45	0.38	0.36	0.47	0.31	0.11	0.22	0.33	0.08
Zhejiang	0.21	0.36	0.45	0.47	0.35	0.51	0.33	0.46	0.42	0.34	0.07	0.36	0.07	0.01
Fujian	0.70	0.29	0.29	0.01	0.55	0.46	0.31	0.31	0.32	0.51	0.21	0.07	0.32	0.06
Shandong	0.45	0.52	0.42	0.25	0.16	0.26	0.16	0.37	0.27	0.06	0.06	0.06	0.47	0.73
Guangdong	0.47	0.60	0.40	0.49	0.29	0.61	0.30	0.60	0.48	0.32	0.19	0.17	0.14	0.40
Hainan	0.31	0.01	0.70	0.14	0.19	0.54	0.58	0.55	0.11	0.56	7.05	0.54	0.72	0.24
Shanxi	0.14	0.30	0.40	0.35	0.27	0.46	0.23	0.07	0.31	0.07	0.08	0.19	0.86	0.23
Anhui	2.57	0.13	0.29	0.14	1.20	0.24	0.20	0.13	0.05	0.14	0.02	0.35	0.01	0.39
Jiangxi	0.46	0.22	0.35	0.05	0.54	0.36	0.13	0.35	0.15	0.41	0.25	0.26	0.33	0.16
Henan	0.06	0.03	0.10	0.27	0.62	0.11	0.09	0.02	0.04	0.13	0.43	0.20	0.08	0.23
Hubei	1.30	0.88	0.80	0.25	0.22	0.15	0.09	0.47	0.18	0.35	0.08	0.27	0.58	0.64
Hunan	0.85	0.21	0.24	0.12	0.40	0.18	0.01	0.14	0.20	0.30	0.12	0.11	0.41	0.41
Inner Mongolia	0.66	0.30	1.13	0.45	0.18	0.22	0.38	0.54	115.00	0.37	0.14	0.12	0.87	1.05
Guangxi	145.67	0.29	0.31	0.14	2.77	0.19	0.17	0.26	0.00	0.36	0.20	0.28	0.20	0.30
Chongqing	0.39	0.21	0.17	0.46	0.50	0.58	0.46	0.29	0.57	0.44	0.39	0.24	0.25	0.37
Sichuan	1.81	0.14	0.04	0.35	0.59	0.40	0.18	0.40	0.37	0.13	0.58	0.38	0.21	0.15
Guizhou	0.78	3.72	0.15	0.86	0.86	0.50	0.54	0.62	0.18	0.23	0.09	0.54	0.28	0.11
Yunnan	0.57	0.21	0.22	0.30	0.64	0.03	0.03	0.42	0.09	0.31	0.19	0.63	0.22	0.23
Tibet	0.07	0.44	1.00	0.11	0.24	0.60	0.92	1.00	0.37	0.21	1.00	0.49	0.57	0.78
Shaanxi	0.38	0.03	0.57	0.05	0.32	0.31	0.06	0.34	0.35	0.56	0.23	0.63	0.26	0.89
Gansu	0.04	0.19	0.10	0.02	0.11	0.09	0.10	0.28	0.25	0.28	0.06	1.05	0.12	11.94
Qinghai	0.50	0.21	1.00	0.59	0.62	0.23	1.00	0.81	0.23	0.78	0.37	0.41	0.52	0.40
Ningxia	0.43	0.42	0.32	0.06	0.16	0.20	0.47	0.18	0.04	0.11	48.67	0.04	0.35	0.30
Xinjiang	0.52	0.76	0.55	0.57	0.32	0.62	0.33	0.62	0.02	0.06	0.54	0.05	0.06	0.42
Liaoning	0.46	0.52	0.03	0.55	0.43	0.14	0.07	0.32	0.24	0.72	1.01	1.00	0.20	1.15
Jilin	0.74	0.72	0.93	0.85	0.86	0.86	0.50	0.60	0.08	0.48	0.62	0.51	0.69	0.16
Heilongjiang	2.53	0.68	1.10	0.87	0.80	0.25	0.57	0.37	0.19	49.67	0.71	0.86	0.50	1.63

**Source:** The authors.

**Table 3:** Time period C_V_ values of 31 provinces (municipalities/autonomous regions) in Mainland China.

Years	2005–2009	2009–2014	2014–2019
Beijing	0.29	0.74	0.88
Tianjin	0.05	0.19	0.82
Hebei	0.02	0.06	0.03
Shanghai	0.34	0.07	1.12
Jiangsu	0.36	0.24	0.23
Zhejiang	0.39	0.14	0.19
Fujian	0.42	0.46	0.29
Shandong	0.45	0.26	0.21
Guangdong	0.20	0.23	0.18
Hainan	0.11	0.48	0.27
Shanxi	0.16	0.15	0.07
Anhui	0.33	0.35	0.08
Jiangxi	0.18	0.35	0.32
Henan	0.06	0.11	0.08
Hubei	0.36	0.11	0.41
Hunan	0.05	0.10	0.03
Inner Mongolia	0.07	0.23	0.05
Guangxi	0.21	0.41	0.30
Chongqing	0.35	0.47	0.15
Sichuan	0.57	0.42	0.32
Guizhou	0.62	0.27	0.31
Yunnan	0.38	0.27	0.09
Tibet	0.27	0.39	0.08
Shaanxi	0.10	0.29	0.43
Gansu	0.07	0.15	0.21
Qinghai	0.27	0.34	0.29
Ningxia	0.34	0.23	0.22
Xinjiang	0.39	0.26	0.07
Liaoning	0.42	0.22	0.90
Jilin	0.83	0.63	0.57
Heilongjiang	0.00	0.49	1.82

**Source:** The authors.

Finally, ArcGIS 10.2 software was used to visualize the imbalance between them (S1 Fig) according to the classification criteria of imbalance between population urbanization and land urbanization (Table 1).

### 4.2. Analysis of measurement results

#### 4.2.1. Analysis of the degree of imbalance

From the perspective of time evolution, the degree of imbalance between population urbanization and land urbanization in China from 2006 to 2019 had slowed down slightly, and the number of provinces (municipalities/autonomous regions) with moderate and above moderate imbalanced urbanization decreased from 19 in 2006 to 16 in 2019. Correspondingly, the number of provinces (municipalities/autonomous regions) with mild and below mild imbalanced urbanization increased from 12 in 2006 to 15 in 2019. Among them, the years with more serious imbalance were 2006, 2008, 2016, 2017, and 2019. In these four years, there were five provinces (municipalities/autonomous regions) experiencing the most serious imbalanced urbanization in 2006, they were Anhui, Hubei, Guangxi, Sichuan, and Heilongjiang while it was extremely imbalanced in Shanghai, Inner Mongolia, Tibet, Qinghai, and Heilongjiang in 2008, and in Shanghai, Hainan, Tibet, Ningxia, and Liaoning in 2016, in Beijing, Tianjin, Shanghai, Gansu, and Liaoning in 2017, and in Beijing, Inner Mongolia, Gansu, Liaoning, and Heilongjiang in 2019. The years with lesser imbalanced urbanization were 2007, 2009, and 2011. In the past three years, only one province (municipality) was in a state of extremely imbalanced urbanization, that is, Guizhou in 2007, Shanghai in 2009 and 2011 (see S1 Fig for details).

By calculating the coefficient of variation of growth over longer periods (Table 3) and comparing it with the assessment criteria in Table 1, the provinces(municipalities) with large changes from light to heavy in their imbalance levels are Beijing, Tianjin, Shanghai, Shaanxi, and Heilongjiang. But Guizhou, Liaoning, and Jilin had great changes from heavy to light. The remaining provinces (municipalities/autonomous regions) had little or no change (S1 Fig).

**Fig 2:**
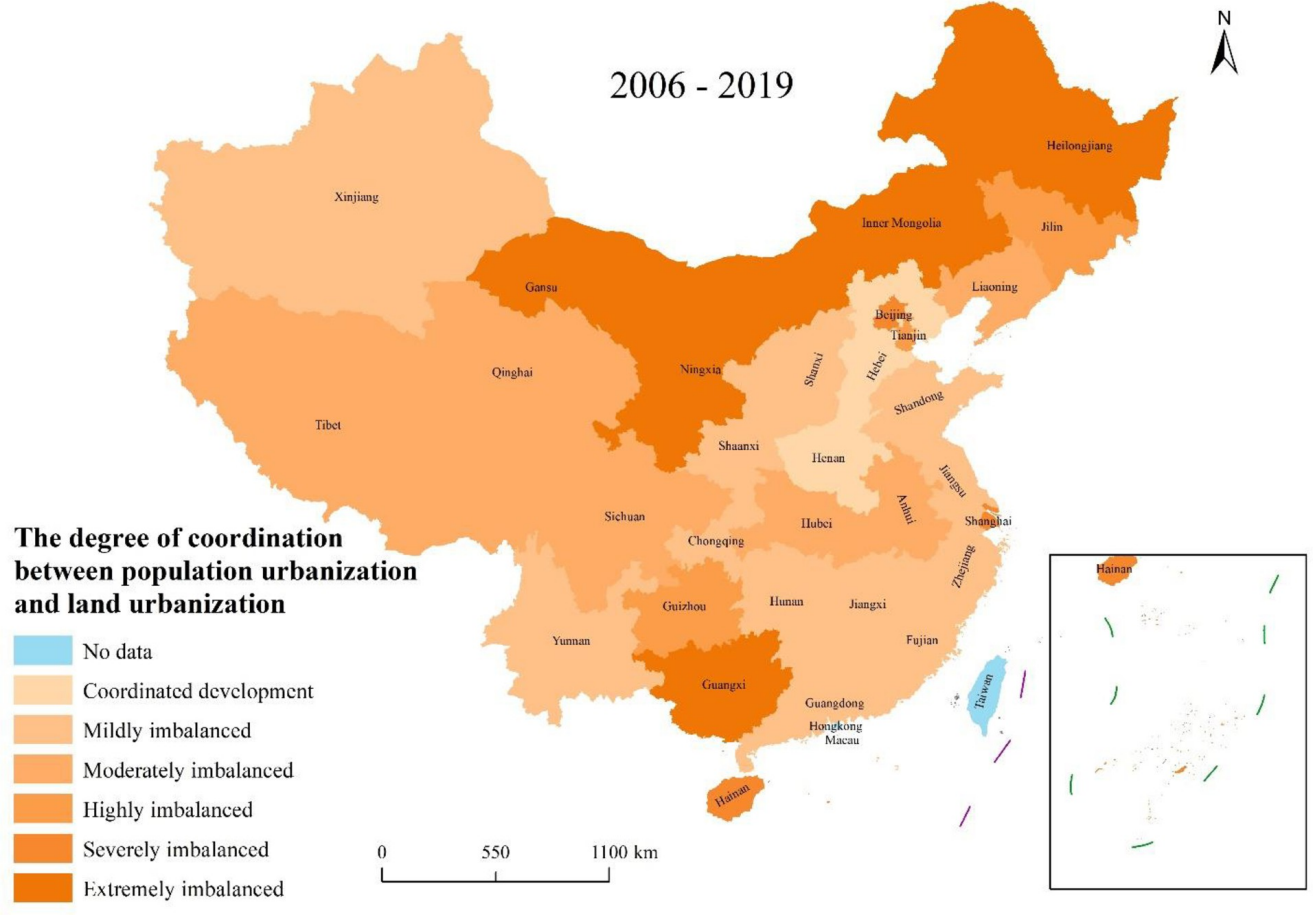
Distribution of imbalance between population urbanization and land urbanization in 31 provinces (municipalities/autonomous regions) of China. **Source:** The authors.

Overall, the coordination of population urbanization and land urbanization in 31 provinces (municipalities/autonomous regions) in the past 14 years is as follows: Hebei and Henan had gained coordinated development as a whole; there was mild imbalanced coordination of population urbanization and land urbanization in 12 provinces (municipalities/autonomous regions) of Jiangsu, Zhejiang, Fujian, Shandong, Guangdong, Shanxi, Jiangxi, Hunan, Chongqing, Yunnan, Shaanxi, and Xinjiang; there was moderate imbalanced coordination in six provinces (autonomous regions) of Anhui, Hubei, Sichuan, Tibet, Qinghai, and Liaoning; in the three provinces (municipalities) of Tianjin, Guizhou, and Jilin, it was highly imbalanced; in the three provinces (municipalities) of Beijing, Shanghai, and Hainan, it was severely imbalanced; and in the five provinces (autonomous regions) of Inner Mongolia, Guangxi, Gansu, Ningxia, and Heilongjiang, it was the most serious imbalanced (Fig 2).

From the spatial distribution point of view, in the past 14 years, the western and northeastern regions had been experiencing serious disorders. Inner Mongolia, Guangxi, Gansu, Ningxia, and Heilongjiang were all at the level of being extremely imbalanced (Fig 2). The coordination in the central region was slightly better, being mildly imbalanced. The disorder in the eastern region was more serious, being moderately imbalanced. Specifically, the degree of imbalance in the east was more serious in 2010, 2016, and 2018, and the degree of imbalance in the remaining years was less serious. The degree of imbalance was more serious in the central region in 2006, which was severely imbalanced, while the degree of imbalance in other years was relatively mild, especially in 2012, 2014, and 2016, which showed a state of coordinated development. The western region was at different levels of disorder during the 14 years from 2006 to 2019, especially in the four years of 2006, 2014, 2016, and 2019, which were extremely imbalanced. The situation in the northeast is similar to that in the west, with varying degrees of imbalance in the 14 years. The nine years of 2006, 2007, 2008, 2009, 2010, 2015, 2016, 2017, and 2019 were all at a highly imbalanced and above grade (see Table 4).

**Table 4:** Average C_V_ and imbalance of population urbanization and land urbanization in four regions of China (2006–2019).

Year	East	Imbalance level	Central	Imbalance level	West	Imbalance level	Northeast	Imbalance level
2006	0.35	Mildly imbalanced	0.90	Severely imbalanced	12.65	Extremely imbalanced	1.24	Extremely imbalanced
2007	0.27	Mildly imbalanced	0.29	Mildly imbalanced	0.58	Moderately imbalanced	0.64	Highly imbalanced
2008	0.47	Moderately imbalanced	0.36	Mildly imbalanced	0.46	Moderately imbalanced	0.69	Highly imbalanced
2009	0.33	Mildly imbalanced	0.20	Mildly imbalanced	0.33	Mildly imbalanced	0.76	Highly imbalanced
2010	0.61	Highly imbalanced	0.54	Moderately imbalanced	0.61	Highly imbalanced	0.70	Highly imbalanced
2011	0.43	Moderately imbalanced	0.25	Mildly imbalanced	0.33	Mildly imbalanced	0.42	Moderately imbalanced
2012	0.37	Mildly imbalanced	0.13	Coordinated development	0.39	Mildly imbalanced	0.38	Mildly imbalanced
2013	0.41	Moderately imbalanced	0.20	Mildly imbalanced	0.48	Moderately imbalanced	0.43	Moderately imbalanced
2014	0.41	Moderately imbalanced	0.16	Coordinated development	9.79	Extremely imbalanced	0.17	Mildly imbalanced
2015	0.39	Mildly imbalanced	0.23	Mildly imbalanced	0.32	Mildly imbalanced	16.95	Extremely imbalanced
2016	1.04	Extremely imbalanced	0.16	Coordinated development	4.37	Extremely imbalanced	0.78	Highly imbalanced
2017	0.47	Moderately imbalanced	0.23	Mildly imbalanced	0.41	Moderately imbalanced	0.79	Highly imbalanced
2018	0.92	Severely imbalanced	0.38	Mildly imbalanced	0.33	Mildly imbalanced	0.46	Moderately imbalanced
2019	0.48	Moderately imbalanced	0.34	Mildly imbalanced	1.41	Extremely imbalanced	0.98	Severely imbalanced
Average	0.50	Moderately imbalanced	0.31	Mildly imbalanced	2.32	Extremely imbalanced	1.81	Extremely imbalanced

**Source:** The authors.

**Fig 3:**
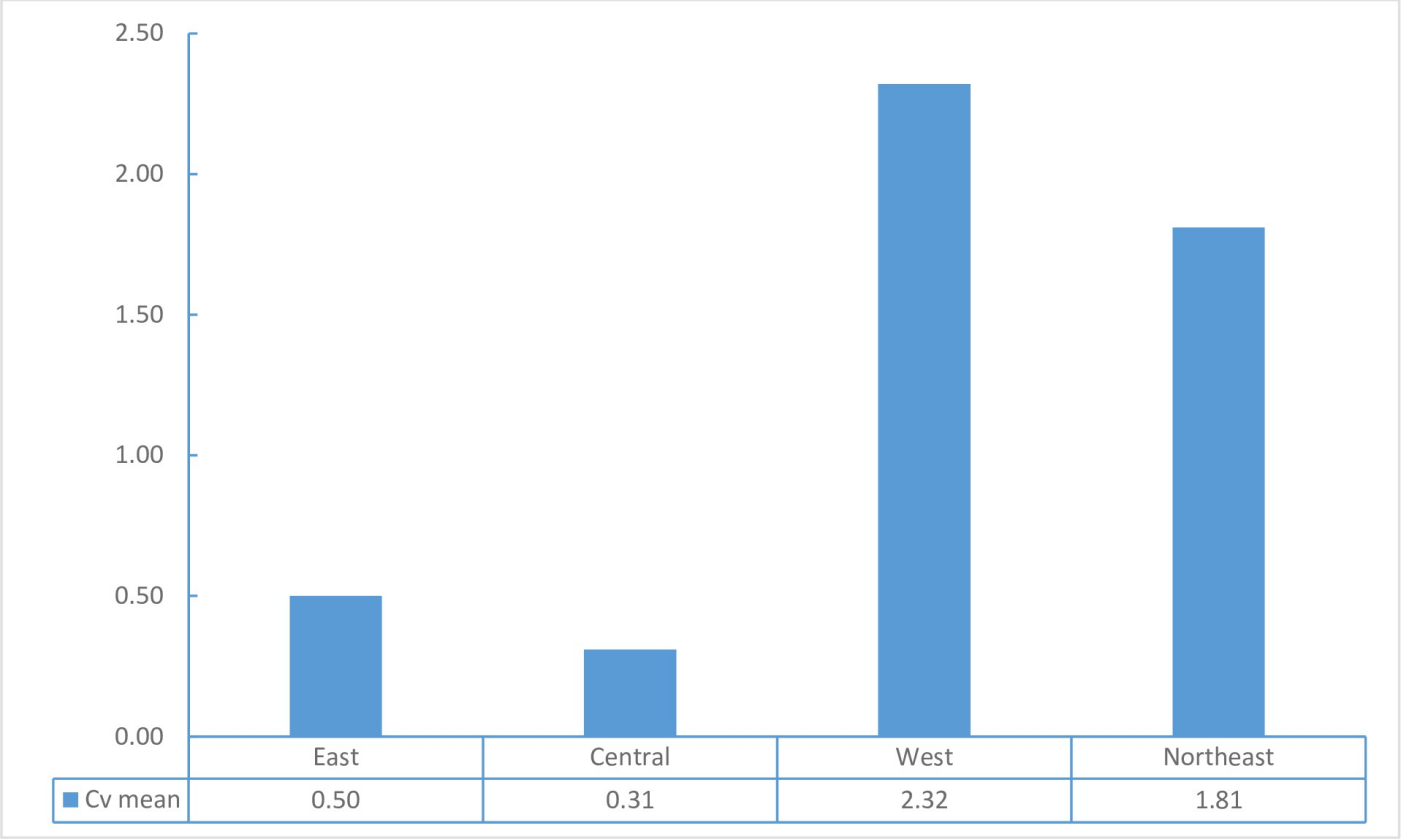
Mean values of C_V_ in the four regions of China. **Source:** The authors.

From the perspective of the average value of the dispersion coefficient, the central region had the lowest degree of imbalance with a dispersion coefficient of 0.31, followed by the eastern region with a dispersion coefficient of 0.50. The northeast and western regions were both the most severe types of imbalance. The magnitude of the value shows that the degree of imbalance in the western region (coefficient of dispersion is 2.32) far exceeded that of the northeast region (coefficient of dispersion is 1.81). The degree of imbalance between population urbanization and land urbanization increased from the central, eastern, northeastern, and western regions (Fig 3).

#### 4.2.2. Analysis of imbalanced types

The coordination of the 10 eastern provinces (municipalities) is as follows: From the perspective of administrative divisions, the remaining eight provinces were all lagging in population urbanization except for Beijing and Hebei, which were lagging in land urbanization. From the perspective of time evolution, they were all lagging in population urbanization every year except for in 2010, in which they were lagging in land urbanization. Overall, the eastern part during the past 14 years fell under the lagging type of population urbanization. The coordination of the six provinces in central China is as follows: from the perspective of administrative divisions, all provinces belonged to the lagging population urbanization type; from the perspective of time evolution, they belonged to the population urbanization lagging type every year except for in 2006 and 2016, in which they are lagging in land urbanization. In the past 14 years, the central part of the country had experienced a lagging type of population urbanization.

The coordination of the 12 western provinces (municipalities/autonomous regions) is as follows: from the perspective of administrative divisions, all belonged to the lagging population urbanization type. From the time series, they were lagging in population urbanization every year except for in 2016 and 2019, in which they were lagging in land urbanization. As a whole, the western region had been lagging behind in population urbanization in the past 14 years.

The coordination of three northeastern provinces is as follows: from the perspective of administrative divisions, they all belonged to the lagging population urbanization type. From the time series, they belonged to the lagging population urbanization type every year except for in 2019, in which they belonged to the land urbanization lagging type. Overall, the northeast had been a lagging population in urbanization over the past 14 years.

**Fig 4:**
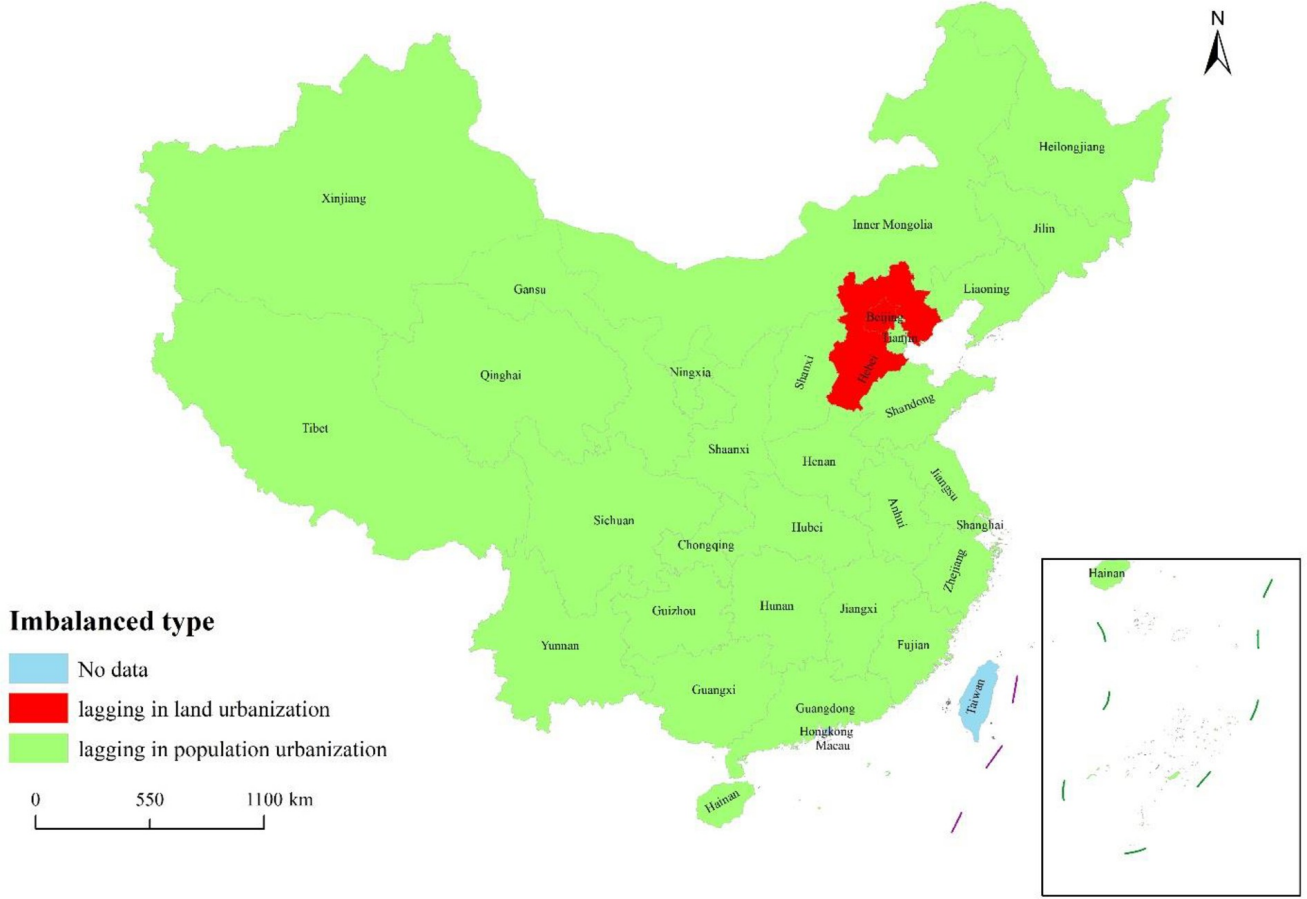
Imbalanced types between population urbanization and land urbanization imbalance in 31 provinces (municipalities/autonomous regions) of Mainland China. **Source:** The authors.

In summary, the population urbanization and land urbanization of 31 provinces (municipalities/autonomous regions) in Mainland China had been seriously imbalanced in the past 14 years, falling under the lagging population urbanization type. From a regional perspective, the degree of imbalance increased in order from central, east, northeast, to the west. The remaining 29 provinces (municipalities/autonomous regions) were generally lagging in population urbanization except for Beijing and Hebei in the east, which were lagging in land urbanization (Fig 4). From the perspective of time, the degree of imbalance in the east was higher in 2010, 2016, and 2018, while the degree of imbalance in other years was relatively small. The imbalance was more serious in the central region in 2006, which was severely imbalanced, while the degree of disorder in other years was relatively mild, especially in 2012, 2014, and 2016, which showed a state of coordinated development. The western region was at varying degrees of imbalance during the 14 years from 2006 to 2019, being extremely imbalanced in the years of 2006, 2014, 2016, and 2019. The situation in the northeast was similar to that in the west, with varying degrees of imbalance during the 14 years. The nine years of 2006, 2007, 2008, 2009, 2010, 2015, 2016, 2017, and 2019 were all at the level of highly imbalanced and above. The regions belonged to the lagging population urbanization type every year except for the central region in 2006, the eastern region in 2010, the central and western regions in 2016, and the western and northeastern regions in 2019, in which they belonged to the lagging land urbanization type.

### 4.3. Discussion of the reasons for the regional and temporal differences between population urbanization and land urbanization in China

#### 4.3.1. Institutional arrangements related to household registration restricting population urbanization

The household registration system in China has experienced a historical evolution from banning cities to severely restricting cities, and then to fully liberalizing cities except for a few megacities. However, the existing system reform has only completed the population from urban and rural imprisonment to free migration and has not fully adjusted the economic interests attached to the household registration. The system associated with the dual household registration—the dual social security system, the dual education system, the dual housing security system—has not cancelled binding with the household registration, and the non-urban population with the household registration are still difficult to enjoy the basic public services of the town [35], which greatly restricts the development of population urbanization.

#### 4.3.2. The dual land system affecting the coordinated development of population and land urbanization

China has a dual land ownership system of collective and state ownership, that is, urban land is owned by the state, and rural and suburban land is owned by the collective unless prescribed by law [36].

First, the land acquisition system affects the coordinated development of population and land urbanization. If it is necessary to occupy collectively owned land in the public interest, it shall be expropriated after examination and approval by the government, the right to use and take ownership of collectively owned land shall be nationalized, and the right to use state-owned land shall be transferred by means of bidding, auction or listing. In the actual operation process, there are defects such as unclear land acquisition purpose, low compensation standard for land expropriation, and difficulty for farmers to enjoy land value-added income, which leads to rapid land urbanization and restricts population urbanization.

Second, the transfer system of contracted land management rights hinders population urbanization. China implements the rural land contract management system [37]. There are two defects of the rural land contract management right transfer system, namely strict limits on the transfer scope of contracted land and unscientific registration system, which increase the transaction costs and opportunity costs of land transfer. However, the unsound agricultural land price evaluation mechanism reduces the compensation of contracted land transfer and leads to the low return of contracted land transfer. In addition to the security function and property function of contracted land [38] and the reality of low income of farmers, farmers have low willingness to transfer contracted land.

Third, the homestead system slows down population urbanization. The restriction the rural homestead system has on population urbanization mainly includes three aspects: strict restriction on the disposal of the homestead, which makes it difficult to realize the most important private property (housing) income of farmers. The regulation that the homestead cannot be mortgaged reduces the ability of peasants to pay for citizenization. And the welfare nature and security function of free allocation and unlimited use of the homestead reduce the willingness of homestead transfer.

#### 4.3.3. The asymmetrical tax distribution system between financial rights and administrative rights affecting the coordinated development of population and land urbanization

In the reform of tax distribution system in our country, there is a problem of "financial power receiving, administrative power moving down". To solve the financial difficulties, the local government can adopt two ways of "reducing expenditure" and "increasing revenue". The reduction of fiscal expenditure will inevitably reduce the supply of public goods and services, as well as the investment and subsidies in education, housing, social security, and infrastructure construction related to people’s livelihood, which will restrict the citizenization of migrant workers. The "income increase" mode of land finance promotes the rapid expansion of land urbanization.

#### 4.3.4. The reasons of more imbalanced western region than eastern region

The reason why the western region is more imbalanced than the eastern region is that the eastern region has fast economic development, more job opportunities, and good social welfare, which attracts a large number of migrants to work and settle in the eastern region. As a result, the population of the eastern region increases rapidly and can better catch up with the rapidly expanding urban land. However, the backward economic development and insufficient supporting facilities lead to a large number of population outflow in the western region, resulting in a more serious degree of uncoordinated development between population urbanization and land urbanization.

#### 4.3.5. The reasons for lagging land urbanization in Beijing and Hebei

The reason why land urbanization lags behind population urbanization in Beijing is because Beijing is China’s political center, cultural center, international exchange center, scientific and technological innovation center, as well as a famous historical and cultural city and one of the ancient capitals. Beijing has the best social hardware, more job opportunities, better welfare conditions, etc., all these result in Beijing being overcrowded. The reason why land urbanization lags behind population urbanization in Hebei is because Hebei is the "back garden" of Beijing, which surrounds the capital. From seamless transportation and ecological protection to industrial transfer and upgrading, the coordinated development of the Beijing-Tianjin-Hebei region has incorporated the whole region into the national strategy. Hebei has made every effort to build itself into first place to relieve non-capital functions of Beijing, focusing on carrying out non-capital functions and population transfer.

## 5. Conclusion

The research on the relationship between population urbanization and land urbanization is a hot issue that scholars have paid close attention to in recent years. The dispersion coefficient of population urbanization and land urbanization from 2006 to 2019 was calculated by using the model with urban population and built-up area indexes, through which the level and type of imbalance between population urbanization and land urbanization are determined. The main conclusions are as follows:

First, in the process of rapid urbanization, population urbanization in most provinces of Mainland China lags behind land urbanization, and population urbanization and land urbanization are in a state of inconsistency. From the perspective of spatial distribution, there are obvious regional differences, which shows a trend of increasing imbalance from central, eastern, northeastern, and western regions, while the imbalance being most serious in the western region.

Second, the author’s preliminary research on the characteristics of population urbanization and land urbanization imbalance in 31 provinces (municipalities/autonomous regions) in Mainland China is helpful in order to understand the temporal and spatial characteristics of population urbanization and land urbanization imbalance at the national level. However, the mechanisms, factors, and countermeasures causing the imbalance between population urbanization and land urbanization have not yet been developed and need to be further discussed in future research.

Third, the research data is mainly from the China Statistical Yearbook. The growth rates of urban population and built-up areas in some provinces and years are negative. This is different from the common understanding. Due to limited personal ability, it is impossible to verify the macro data. In future research, data acquisition and model measurements need to be continuously explored and improved.

Finally, the regional differences between China’s population urbanization and land urbanization are mainly caused by China’s dual household registration system, dual land system and the asymmetrical tax distribution system between financial rights and administrative rights.

## Supporting information

S1 Appendix Urban population and built-up area of 31 provinces (municipalities/autonomous regions) in Mainland China (2005–2019). Unit: 10^4^people / km^2^. **Source:** China Statistical Yearbook. **Notes:** BA represents built-up area and UP represents urban population.
(DOCX)

S2 Appendix P value of 31 provinces (municipalities/autonomous regions) in Mainland China (2006–2019). Unit: %. **Source:** The authors. **Note:** P is the growth rate of population in urban areas.
(DOCX)

S3 Appendix L value of 31 provinces (municipalities/autonomous regions) in Mainland China (2006–2019). Unit: %**. Source:** The authors. **Note:** L is the growth rate of the built-up area.
(DOCX)

S1 Fig China’s population urbanization and land urbanization imbalance distribution. **Source:** The authors.
(DOCX)
