# Supplementary material for: A regional comparative study on the mismatch between population urbanization and land urbanization in China
Source: PLoS One. 2023 Jun 30;18(6):e0287366. doi: 10.1371/journal.pone.0287366 (PMC10313039; doi:10.1371/journal.pone.0287366)
Supplement: S1 Appendix — Unit: 104people / km2. Source: China Statistical Yearbook. Notes: BA represents built-up area and UP represents urban population. (DOCX) [file pone.0287366.s001.docx]

**Appendix 1.** Urban population and built-up area of 31 provinces (municipalities/autonomous regions) in Mainland China (2005–2019) Unit: 10^4^people / km^2^

| Area | Index | 2005 | 2006 | 2007 | 2008 | 2009 | 2010 | 2011 | 2012 | 2013 | 2014 | 2015 | 2016 | 2017 | 2018 | 2019 |
| --- | --- | --- | --- | --- | --- | --- | --- | --- | --- | --- | --- | --- | --- | --- | --- | --- |
| Beijing | UP | 1286 | 1350 | 1416 | 1504 | 1581 | 1686 | 1740 | 1784 | 1825 | 1858 | 1877 | 1880 | 1878 | 1863 | 1865 |
|  | BA | 1200 | 1254 | 1289 | 1311 | 1350 | 1186 | 1231 | 1261 | 1306 | 1386 | 1401 | 1420 | 1446 | 1469 | 1469 |
| Tianjin | UP | 783 | 814 | 851 | 908 | 958 | 1034 | 1090 | 1152 | 1207 | 1248 | 1278 | 1295 | 1291 | 1297 | 1304 |
|  | BA | 530 | 540 | 572 | 641 | 662 | 687 | 711 | 722 | 747 | 797 | 885 | 1008 | 1088 | 1078 | 1151 |
| Hebei | UP | 2582 | 2674 | 2795 | 2928 | 3077 | 3201 | 3302 | 3411 | 3528 | 3642 | 3811 | 3983 | 4136 | 4264 | 4374 |
|  | BA | 1316 | 1417 | 1474 | 1528 | 1578 | 1620 | 1685 | 1739 | 1787 | 1833 | 1944 | 2056 | 2120 | 2163 | 2182 |
| Shanghai | UP | 1684 | 1742 | 1830 | 1897 | 1958 | 2056 | 2096 | 2126 | 2164 | 2173 | 2116 | 2127 | 2121 | 2136 | 2144 |
|  | BA | 820 | 860 | 886 | 886 | 886 | 999 | 999 | 999 | 999 | 999 | 999 | 999 | 999 | 1238 | 1238 |
| Jiangsu | UP | 3832 | 3973 | 4109 | 4215 | 4343 | 4767 | 4889 | 4990 | 5090 | 5191 | 5306 | 5417 | 5521 | 5604 | 5698 |
|  | BA | 2379 | 2583 | 2714 | 2904 | 3046 | 3271 | 3494 | 3655 | 3810 | 4020 | 4189 | 4299 | 4427 | 4558 | 4648 |
| Zhejiang | UP | 2796 | 2866 | 2949 | 3002 | 3055 | 3356 | 3403 | 3461 | 3519 | 3573 | 3645 | 3745 | 3847 | 3953 | 4095 |
|  | BA | 1680 | 1744 | 1851 | 1939 | 2033 | 2129 | 2221 | 2296 | 2399 | 2489 | 2591 | 2673 | 2829 | 2919 | 3022 |
| Fujian | UP | 1757 | 1807 | 1857 | 1929 | 2020 | 2109 | 2161 | 2234 | 2293 | 2352 | 2403 | 2464 | 2534 | 2594 | 2642 |
|  | BA | 673 | 780 | 819 | 877 | 919 | 1059 | 1130 | 1203 | 1263 | 1326 | 1414 | 1469 | 1517 | 1588 | 1621 |
| Shandong | UP | 4162 | 4291 | 4379 | 4483 | 4576 | 4765 | 4910 | 5078 | 5232 | 5385 | 5614 | 5871 | 6062 | 6147 | 6194 |
|  | BA | 2676 | 2895 | 3082 | 3261 | 3374 | 3566 | 3751 | 3927 | 4187 | 4400 | 4609 | 4795 | 4971 | 5164 | 5413 |
| Guangdong | UP | 5579 | 5949 | 6099 | 6269 | 6423 | 6910 | 6986 | 7140 | 7212 | 7292 | 7454 | 7611 | 7802 | 8022 | 8226 |
|  | BA | 3619 | 3706 | 4084 | 4133 | 4434 | 4618 | 4829 | 5026 | 5232 | 5398 | 5633 | 5808 | 5911 | 6036 | 6398 |
| Hainan | UP | 374 | 385 | 399 | 410 | 425 | 433 | 443 | 457 | 472 | 486 | 502 | 521 | 537 | 552 | 560 |
|  | BA | 194 | 197 | 204 | 205 | 215 | 221 | 238 | 266 | 296 | 303 | 338 | 321 | 324 | 380 | 383 |
| Shanxi | UP | 1413 | 1451 | 1494 | 1539 | 1576 | 1717 | 1785 | 1851 | 1908 | 1962 | 2016 | 2070 | 2123 | 2172 | 2221 |
|  | BA | 709 | 734 | 774 | 784 | 823 | 865 | 957 | 1014 | 1041 | 1097 | 1123 | 1158 | 1178 | 1180 | 1223 |
| Anhui | UP | 2173 | 2267 | 2368 | 2485 | 2581 | 2562 | 2674 | 2784 | 2886 | 2990 | 3103 | 3221 | 3346 | 3459 | 3553 |
|  | BA | 1260 | 1136 | 1202 | 1311 | 1378 | 1491 | 1598 | 1696 | 1777 | 1835 | 1926 | 2002 | 2039 | 2110 | 2242 |
| Jiangxi | UP | 1595 | 1678 | 1739 | 1820 | 1914 | 1966 | 2051 | 2140 | 2210 | 2281 | 2357 | 2438 | 2524 | 2604 | 2679 |
|  | BA | 664 | 758 | 801 | 819 | 857 | 934 | 1020 | 1078 | 1151 | 1201 | 1296 | 1371 | 1454 | 1546 | 1608 |
| Henan | UP | 2875 | 3050 | 3214 | 3397 | 3577 | 3621 | 3809 | 3991 | 4123 | 4265 | 4441 | 4623 | 4795 | 4967 | 5129 |
|  | BA | 1572 | 1679 | 1775 | 1857 | 1913 | 2014 | 2098 | 2219 | 2289 | 2375 | 2503 | 2544 | 2685 | 2797 | 2944 |
| Hubei | UP | 2467 | 2494 | 2525 | 2581 | 2631 | 2847 | 2984 | 3092 | 3161 | 3238 | 3327 | 3419 | 3500 | 3568 | 3615 |
|  | BA | 1417 | 1298 | 1299 | 1565 | 1616 | 1701 | 1812 | 1890 | 2007 | 2078 | 2197 | 2249 | 2341 | 2510 | 2661 |
| Hunan | UP | 2341 | 2455 | 2571 | 2689 | 2767 | 2845 | 2975 | 3097 | 3209 | 3320 | 3452 | 3599 | 3747 | 3865 | 3959 |
|  | BA | 1033 | 1037 | 1112 | 1195 | 1239 | 1321 | 1408 | 1465 | 1505 | 1540 | 1573 | 1626 | 1709 | 1837 | 1856 |
| Inner Mongolia | UP | 1134 | 1175 | 1218 | 1264 | 1313 | 1372 | 1405 | 1438 | 1466 | 1491 | 1514 | 1542 | 1568 | 1589 | 1609 |
|  | BA | 824 | 830 | 887 | 885 | 975 | 1038 | 1077 | 1133 | 1206 | 1185 | 1225 | 1242 | 1269 | 1270 | 1270 |
| Guangxi | UP | 1567 | 1635 | 1728 | 1838 | 1904 | 1844 | 1942 | 2038 | 2115 | 2187 | 2257 | 2326 | 2404 | 2474 | 2534 |
|  | BA | 772 | 738 | 814 | 841 | 881 | 940 | 1014 | 1084 | 1154 | 1193 | 1275 | 1334 | 1414 | 1476 | 1543 |
| Chongqing | UP | 1265 | 1311 | 1360 | 1419 | 1475 | 1529 | 1606 | 1678 | 1733 | 1783 | 1838 | 1908 | 1971 | 2032 | 2087 |
|  | BA | 583 | 631 | 667 | 708 | 783 | 870 | 1035 | 1052 | 1115 | 1231 | 1329 | 1351 | 1423 | 1497 | 1515 |
| Sichuan | UP | 2710 | 2802 | 2893 | 3044 | 3168 | 3232 | 3367 | 3516 | 3640 | 3769 | 3912 | 4066 | 4217 | 4362 | 4505 |
|  | BA | 1443 | 1273 | 1328 | 1392 | 1510 | 1630 | 1788 | 1902 | 2058 | 2217 | 2282 | 2616 | 2832 | 2982 | 3054 |
| Guizhou | UP | 1002 | 1013 | 1026 | 1047 | 1057 | 1176 | 1213 | 1269 | 1325 | 1404 | 1483 | 1570 | 1648 | 1711 | 1776 |
|  | BA | 372 | 405 | 396 | 407 | 460 | 464 | 508 | 586 | 695 | 724 | 789 | 845 | 986 | 1053 | 1086 |
| Yunnan | UP | 1313 | 1367 | 1426 | 1499 | 1554 | 1597 | 1704 | 1831 | 1897 | 1967 | 2055 | 2148 | 2241 | 2309 | 2376 |
|  | BA | 472 | 542 | 578 | 624 | 667 | 751 | 804 | 860 | 936 | 977 | 1060 | 1131 | 1142 | 1164 | 1218 |
| Tibet | UP | 58 | 60 | 62 | 64 | 66 | 68 | 69 | 70 | 74 | 82 | 90 | 98 | 104 | 107 | 111 |
|  | BA | 75 | 78 | 79 | 79 | 81 | 85 | 90 | 120 | 120 | 126 | 145 | 145 | 148 | 164 | 164 |
| Shaanxi | UP | 1374 | 1447 | 1506 | 1565 | 1621 | 1709 | 1770 | 1877 | 1931 | 1985 | 2045 | 2110 | 2178 | 2246 | 2304 |
|  | BA | 562 | 629 | 653 | 660 | 686 | 758 | 809 | 864 | 915 | 968 | 1073 | 1127 | 1287 | 1356 | 1358 |
| Gansu | UP | 764 | 792 | 822 | 856 | 891 | 925 | 953 | 999 | 1036 | 1080 | 1123 | 1166 | 1218 | 1258 | 1284 |
|  | BA | 507 | 524 | 553 | 581 | 604 | 633 | 656 | 682 | 727 | 779 | 834 | 870 | 869 | 891 | 876 |
| Qinghai | UP | 213 | 215 | 221 | 226 | 234 | 252 | 263 | 272 | 280 | 290 | 296 | 306 | 317 | 328 | 337 |
|  | BA | 106 | 109 | 111 | 111 | 112 | 114 | 122 | 122 | 157 | 166 | 194 | 197 | 200 | 202 | 215 |
| Ningxia | UP | 252 | 260 | 269 | 278 | 288 | 303 | 319 | 328 | 340 | 355 | 369 | 380 | 395 | 405 | 416 |
|  | BA | 249 | 269 | 292 | 311 | 321 | 344 | 371 | 400 | 421 | 441 | 455 | 442 | 458 | 482 | 489 |
| Xinjiang | UP | 747 | 778 | 820 | 845 | 860 | 940 | 962 | 982 | 1007 | 1059 | 1115 | 1159 | 1207 | 1266 | 1309 |
|  | BA | 596 | 674 | 679 | 751 | 800 | 838 | 922 | 960 | 1065 | 1118 | 1185 | 1199 | 1244 | 1312 | 1422 |
| Liaoning | UP | 2478 | 2519 | 2544 | 2591 | 2620 | 2717 | 2807 | 2881 | 2917 | 2944 | 2952 | 2949 | 2949 | 2968 | 2964 |
|  | BA | 1780 | 1860 | 1918 | 1956 | 2031 | 2221 | 2277 | 2329 | 2386 | 2422 | 2462 | 2798 | 2644 | 2670 | 2720 |
| Jilin | UP | 1426 | 1442 | 1451 | 1455 | 1461 | 1465 | 1468 | 1477 | 1491 | 1509 | 1523 | 1530 | 1539 | 1556 | 1568 |
|  | BA | 943 | 1013 | 1051 | 1135 | 1193 | 1237 | 1271 | 1294 | 1344 | 1363 | 1399 | 1426 | 1452 | 1539 | 1555 |
| Heilongjiang | UP | 2028 | 2045 | 2061 | 2119 | 2123 | 2134 | 2166 | 2182 | 2201 | 2224 | 2241 | 2249 | 2250 | 2268 | 2284 |
|  | BA | 1496 | 1467 | 1526 | 1524 | 1566 | 1638 | 1679 | 1725 | 1758 | 1785 | 1772 | 1810 | 1820 | 1825 | 1771 |

**Source:** China Statistical Yearbook.

**Notes:** BA represents built-up area and UP represents urban population.
