# Supplementary material for: A regional comparative study on the mismatch between population urbanization and land urbanization in China
Source: PLoS One. 2023 Jun 30;18(6):e0287366. doi: 10.1371/journal.pone.0287366 (PMC10313039; doi:10.1371/journal.pone.0287366)
Supplement: S2 Appendix — Unit: %. Source: The authors. Note: P is the growth rate of population in urban areas. (DOCX) [file pone.0287366.s002.docx]

**Appendix 2.** P value of 31 provinces (municipalities/autonomous regions) in Mainland China (2006–2019) Unit: %

| **Years** | **2006** | **2007** | **2008** | **2009** | **2010** | **2011** | **2012** | **2013** | **2014** | **2015** | **2016** | **2017** | **2018** | **2019** |
| --- | --- | --- | --- | --- | --- | --- | --- | --- | --- | --- | --- | --- | --- | --- |
| Beijing | 4.98 | 4.89 | 6.21 | 5.12 | 6.64 | 3.20 | 2.53 | 2.30 | 1.81 | 1.02 | 0.16 | -0.11 | -0.80 | 0.11 |
| Tianjin | 3.96 | 4.55 | 6.70 | 5.51 | 7.93 | 5.42 | 5.69 | 4.77 | 3.40 | 2.40 | 1.33 | -0.31 | 0.46 | 0.54 |
| Hebei | 3.56 | 4.53 | 4.76 | 5.09 | 4.03 | 3.16 | 3.30 | 3.43 | 3.23 | 4.64 | 4.51 | 3.84 | 3.09 | 2.58 |
| Shanghai | 3.44 | 5.05 | 3.66 | 3.22 | 5.01 | 1.95 | 1.43 | 1.79 | 0.42 | -2.62 | 0.52 | -0.28 | 0.71 | 0.37 |
| Jiangsu | 3.68 | 3.42 | 2.58 | 3.04 | 9.76 | 2.56 | 2.07 | 2.00 | 1.98 | 2.22 | 2.09 | 1.92 | 1.50 | 1.68 |
| Zhejiang | 2.50 | 2.90 | 1.80 | 1.77 | 9.85 | 1.40 | 1.70 | 1.68 | 1.53 | 2.02 | 2.74 | 2.72 | 2.76 | 3.59 |
| Fujian | 2.85 | 2.77 | 3.88 | 4.72 | 4.41 | 2.47 | 3.38 | 2.64 | 2.57 | 2.17 | 2.54 | 2.84 | 2.37 | 1.85 |
| Shandong | 3.10 | 2.05 | 2.37 | 2.07 | 4.13 | 3.04 | 3.42 | 3.03 | 2.92 | 4.25 | 4.58 | 3.25 | 1.40 | 0.76 |
| Guangdong | 6.63 | 2.52 | 2.79 | 2.46 | 7.58 | 1.10 | 2.20 | 1.01 | 1.11 | 2.22 | 2.11 | 2.51 | 2.82 | 2.54 |
| Hainan | 2.94 | 3.64 | 2.76 | 3.66 | 1.88 | 2.31 | 3.16 | 3.28 | 2.97 | 3.29 | 3.78 | 3.07 | 2.79 | 1.45 |
| Shanxi | 2.69 | 2.96 | 3.01 | 2.40 | 8.95 | 3.96 | 3.70 | 3.08 | 2.83 | 2.75 | 2.68 | 2.56 | 2.31 | 2.26 |
| Anhui | 4.33 | 4.46 | 4.94 | 3.86 | -0.74 | 4.37 | 4.11 | 3.66 | 3.60 | 3.78 | 3.80 | 3.88 | 3.38 | 2.72 |
| Jiangxi | 5.20 | 3.64 | 4.66 | 5.16 | 2.72 | 4.32 | 4.34 | 3.27 | 3.21 | 3.33 | 3.44 | 3.53 | 3.17 | 2.88 |
| Henan | 6.09 | 5.38 | 5.69 | 5.30 | 1.23 | 5.19 | 4.78 | 3.31 | 3.44 | 4.13 | 4.10 | 3.72 | 3.59 | 3.26 |
| Hubei | 1.09 | 1.24 | 2.22 | 1.94 | 8.21 | 4.81 | 3.62 | 2.23 | 2.44 | 2.75 | 2.77 | 2.37 | 1.94 | 1.32 |
| Hunan | 4.87 | 4.73 | 4.59 | 2.90 | 2.82 | 4.57 | 4.10 | 3.62 | 3.46 | 3.98 | 4.26 | 4.11 | 3.15 | 2.43 |
| Inner Mongolia | 3.62 | 3.66 | 3.78 | 3.88 | 4.49 | 2.41 | 2.35 | 1.95 | 1.71 | 1.54 | 1.85 | 1.69 | 1.34 | 1.26 |
| Guangxi | 4.34 | 5.69 | 6.37 | 3.59 | -3.15 | 5.31 | 4.94 | 3.78 | 3.40 | 3.20 | 3.06 | 3.35 | 2.91 | 2.43 |
| Chongqing | 3.64 | 3.74 | 4.34 | 3.95 | 3.66 | 5.04 | 4.48 | 3.28 | 2.89 | 3.08 | 3.81 | 3.30 | 3.09 | 2.71 |
| Sichuan | 3.39 | 3.25 | 5.22 | 4.07 | 2.02 | 4.18 | 4.43 | 3.53 | 3.54 | 3.79 | 3.94 | 3.71 | 3.44 | 3.28 |
| Guizhou | 1.10 | 1.28 | 2.05 | 0.96 | 11.26 | 3.15 | 4.62 | 4.41 | 5.96 | 5.63 | 5.87 | 4.97 | 3.82 | 3.80 |
| Yunnan | 4.11 | 4.32 | 5.12 | 3.67 | 2.77 | 6.70 | 7.45 | 3.60 | 3.69 | 4.47 | 4.53 | 4.33 | 3.03 | 2.90 |
| Tibet | 3.45 | 3.33 | 3.23 | 3.13 | 3.03 | 1.47 | 1.45 | 5.71 | 10.81 | 9.76 | 8.89 | 6.12 | 2.88 | 3.74 |
| Shaanxi | 5.31 | 4.08 | 3.92 | 3.58 | 5.43 | 3.57 | 6.05 | 2.88 | 2.80 | 3.02 | 3.18 | 3.22 | 3.12 | 2.58 |
| Gansu | 3.66 | 3.79 | 4.14 | 4.09 | 3.82 | 3.03 | 4.83 | 3.70 | 4.25 | 3.98 | 3.83 | 4.46 | 3.28 | 2.07 |
| Qinghai | 0.94 | 2.79 | 2.26 | 3.54 | 7.69 | 4.37 | 3.42 | 2.94 | 3.57 | 2.07 | 3.38 | 3.59 | 3.47 | 2.74 |
| Ningxia | 3.17 | 3.46 | 3.35 | 3.60 | 5.21 | 5.28 | 2.82 | 3.66 | 4.41 | 3.94 | 2.98 | 3.95 | 2.53 | 2.72 |
| Xinjiang | 4.15 | 5.40 | 3.05 | 1.78 | 9.30 | 2.34 | 2.08 | 2.55 | 5.16 | 5.29 | 3.95 | 4.14 | 4.89 | 3.40 |
| Liaoning | 1.65 | 0.99 | 1.85 | 1.12 | 3.70 | 3.31 | 2.64 | 1.25 | 0.93 | 0.27 | -0.10 | 0.00 | 0.64 | -0.13 |
| Jilin | 1.12 | 0.62 | 0.28 | 0.41 | 0.27 | 0.20 | 0.61 | 0.95 | 1.21 | 0.93 | 0.46 | 0.59 | 1.10 | 0.77 |
| Heilongjiang | 0.84 | 0.78 | 2.81 | 0.19 | 0.52 | 1.50 | 0.74 | 0.87 | 1.04 | 0.76 | 0.36 | 0.04 | 0.80 | 0.71 |

**Source:** The authors.

**Note:** P is the growth rate of population in urban areas.
