# Supplementary material for: A regional comparative study on the mismatch between population urbanization and land urbanization in China
Source: PLoS One. 2023 Jun 30;18(6):e0287366. doi: 10.1371/journal.pone.0287366 (PMC10313039; doi:10.1371/journal.pone.0287366)
Supplement: S3 Appendix — Unit: %. Source: The authors. Note: L is the growth rate of the built-up area. (DOCX) [file pone.0287366.s003.docx]

**Appendix 3.** L value of 31 provinces (municipalities/autonomous regions) in Mainland China (2006–2019) Unit: %

| **Years** | **2006** | **2007** | **2008** | **2009** | **2010** | **2011** | **2012** | **2013** | **2014** | **2015** | **2016** | **2017** | **2018** | **2019** |
| --- | --- | --- | --- | --- | --- | --- | --- | --- | --- | --- | --- | --- | --- | --- |
| Beijing | 4.50 | 2.79 | 1.71 | 2.97 | -12.15 | 3.79 | 2.44 | 3.57 | 6.13 | 1.08 | 1.36 | 1.83 | 1.59 | 0.00 |
| Tianjin | 1.89 | 5.93 | 12.06 | 3.28 | 3.78 | 3.49 | 1.55 | 3.46 | 6.69 | 11.04 | 13.90 | 7.94 | -0.93 | 6.79 |
| Hebei | 7.67 | 4.02 | 3.66 | 3.27 | 2.66 | 4.01 | 3.20 | 2.76 | 2.57 | 6.06 | 5.76 | 3.11 | 2.02 | 0.90 |
| Shanghai | 4.88 | 3.02 | 0.00 | 0.00 | 12.75 | 0.00 | 0.00 | 0.00 | 0.00 | 0.00 | 0.00 | 0.00 | 23.90 | 0.01 |
| Jiangsu | 8.58 | 5.07 | 7.00 | 4.89 | 7.39 | 6.82 | 4.61 | 4.24 | 5.51 | 4.20 | 2.63 | 2.98 | 2.97 | 1.97 |
| Zhejiang | 3.81 | 6.14 | 4.75 | 4.85 | 4.72 | 4.32 | 3.38 | 4.49 | 3.75 | 4.10 | 3.16 | 5.84 | 3.18 | 3.52 |
| Fujian | 15.90 | 5.00 | 7.08 | 4.79 | 15.23 | 6.70 | 6.46 | 4.99 | 4.99 | 6.64 | 3.89 | 3.27 | 4.65 | 2.09 |
| Shandong | 8.18 | 6.46 | 5.81 | 3.47 | 5.69 | 5.19 | 4.69 | 6.62 | 5.09 | 4.75 | 4.04 | 3.67 | 3.89 | 4.81 |
| Guangdong | 2.40 | 10.20 | 1.20 | 7.28 | 4.15 | 4.57 | 4.08 | 4.10 | 3.17 | 4.35 | 3.11 | 1.77 | 2.12 | 5.99 |
| Hainan | 1.55 | 3.55 | 0.49 | 4.88 | 2.79 | 7.69 | 11.76 | 11.28 | 2.36 | 11.55 | -5.03 | 0.93 | 17.14 | 0.88 |
| Shanxi | 3.53 | 5.45 | 1.29 | 4.97 | 5.10 | 10.64 | 5.96 | 2.66 | 5.38 | 2.37 | 3.12 | 1.73 | 0.18 | 3.61 |
| Anhui | -9.84 | 5.81 | 9.07 | 5.11 | 8.20 | 7.18 | 6.13 | 4.78 | 3.26 | 4.96 | 3.95 | 1.85 | 3.48 | 6.24 |
| Jiangxi | 14.16 | 5.67 | 2.25 | 4.64 | 8.98 | 9.21 | 5.69 | 6.77 | 4.34 | 7.91 | 5.79 | 6.05 | 6.35 | 3.98 |
| Henan | 6.81 | 5.72 | 4.62 | 3.02 | 5.28 | 4.17 | 5.77 | 3.15 | 3.76 | 5.39 | 1.64 | 5.54 | 4.18 | 5.25 |
| Hubei | -8.40 | 0.08 | 20.48 | 3.26 | 5.26 | 6.53 | 4.30 | 6.19 | 3.54 | 5.73 | 2.37 | 4.09 | 7.21 | 6.03 |
| Hunan | 0.39 | 7.23 | 7.46 | 3.68 | 6.62 | 6.59 | 4.05 | 2.73 | 2.33 | 2.14 | 3.37 | 5.10 | 7.50 | 1.02 |
| Inner Mongolia | 0.73 | 6.87 | -0.23 | 10.17 | 6.46 | 3.76 | 5.20 | 6.44 | -1.74 | 3.38 | 1.39 | 2.17 | 0.09 | -0.03 |
| Guangxi | -4.40 | 10.30 | 3.32 | 4.76 | 6.70 | 7.87 | 6.90 | 6.46 | 3.38 | 6.87 | 4.63 | 6.00 | 4.38 | 4.53 |
| Chongqing | 8.23 | 5.71 | 6.15 | 10.59 | 11.11 | 18.97 | 1.64 | 5.99 | 10.40 | 7.96 | 1.66 | 5.33 | 5.18 | 1.25 |
| Sichuan | -11.78 | 4.32 | 4.82 | 8.48 | 7.95 | 9.69 | 6.38 | 8.20 | 7.73 | 2.93 | 14.64 | 8.26 | 5.31 | 2.41 |
| Guizhou | 8.87 | -2.22 | 2.78 | 13.02 | 0.87 | 9.48 | 15.35 | 18.60 | 4.17 | 8.98 | 7.10 | 16.69 | 6.81 | 3.07 |
| Yunnan | 14.83 | 6.64 | 7.96 | 6.89 | 12.59 | 7.06 | 6.97 | 8.84 | 4.38 | 8.50 | 6.70 | 0.97 | 1.92 | 4.61 |
| Tibet | 4.00 | 1.28 | 0.00 | 2.53 | 4.94 | 5.88 | 33.33 | 0.00 | 5.00 | 15.08 | 0.00 | 2.07 | 10.59 | 0.46 |
| Shaanxi | 11.92 | 3.82 | 1.07 | 3.94 | 10.50 | 6.73 | 6.80 | 5.90 | 5.79 | 10.85 | 5.03 | 14.20 | 5.32 | 0.15 |
| Gansu | 3.35 | 5.53 | 5.06 | 3.96 | 4.80 | 3.63 | 3.96 | 6.60 | 7.15 | 7.06 | 4.32 | -0.11 | 2.57 | -1.75 |
| Qinghai | 2.83 | 1.83 | 0.00 | 0.90 | 1.79 | 7.02 | 0.00 | 28.69 | 5.73 | 16.87 | 1.55 | 1.52 | 1.11 | 6.41 |
| Ningxia | 8.03 | 8.55 | 6.51 | 3.22 | 7.17 | 7.85 | 7.82 | 5.25 | 4.75 | 3.17 | -2.86 | 3.62 | 5.24 | 1.46 |
| Xinjiang | 13.09 | 0.74 | 10.60 | 6.52 | 4.75 | 10.02 | 4.12 | 10.94 | 4.98 | 5.99 | 1.18 | 3.75 | 5.49 | 8.34 |
| Liaoning | 4.49 | 3.12 | 1.98 | 3.83 | 9.35 | 2.52 | 2.28 | 2.45 | 1.51 | 1.65 | 13.65 | -5.50 | 0.97 | 1.89 |
| Jilin | 7.42 | 3.75 | 7.99 | 5.11 | 3.69 | 2.75 | 1.81 | 3.86 | 1.41 | 2.64 | 1.93 | 1.82 | 5.98 | 1.06 |
| Heilongjiang | -1.94 | 4.02 | -0.13 | 2.76 | 4.60 | 2.50 | 2.74 | 1.91 | 1.54 | -0.73 | 2.14 | 0.55 | 0.27 | -2.96 |

**Source:** The authors.

**Note:** L is the growth rate of the built-up area.
