# Supplementary material for: A regional comparative study on the mismatch between population urbanization and land urbanization in China
Source: PLoS One. 2023 Jun 30;18(6):e0287366. doi: 10.1371/journal.pone.0287366 (PMC10313039; doi:10.1371/journal.pone.0287366)
Supplement: S1 Fig — Source: The authors. (DOCX) [file pone.0287366.s004.docx]

**S1 Fig: China's population urbanization and land urbanization imbalance distribution**

**Source:** The authors.


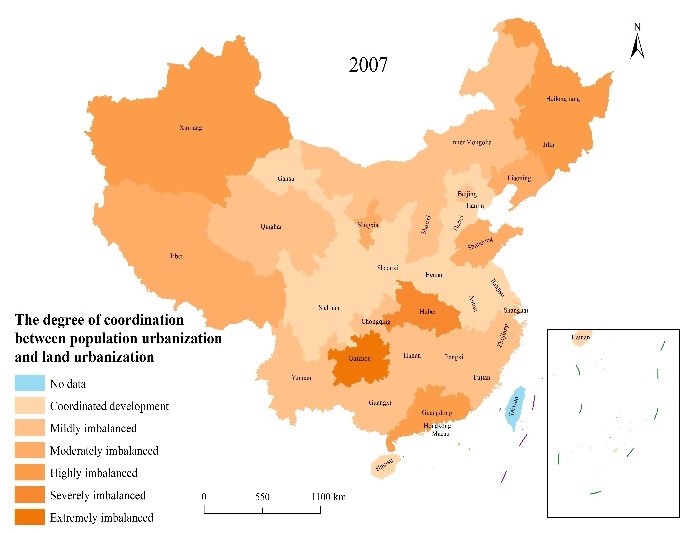

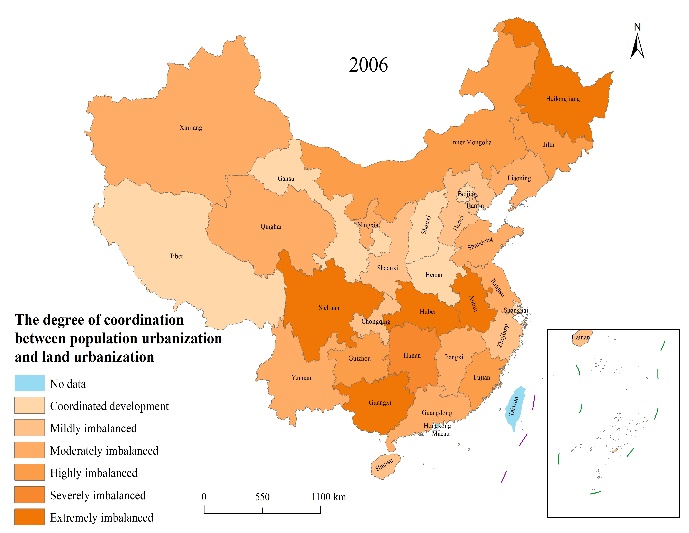

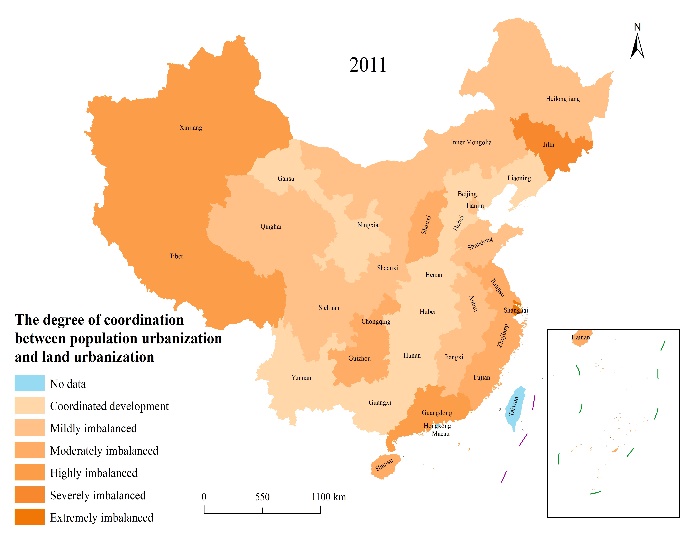

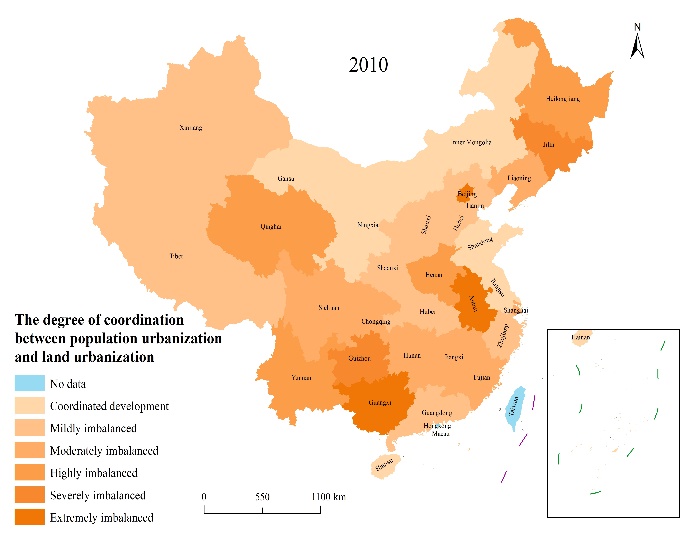

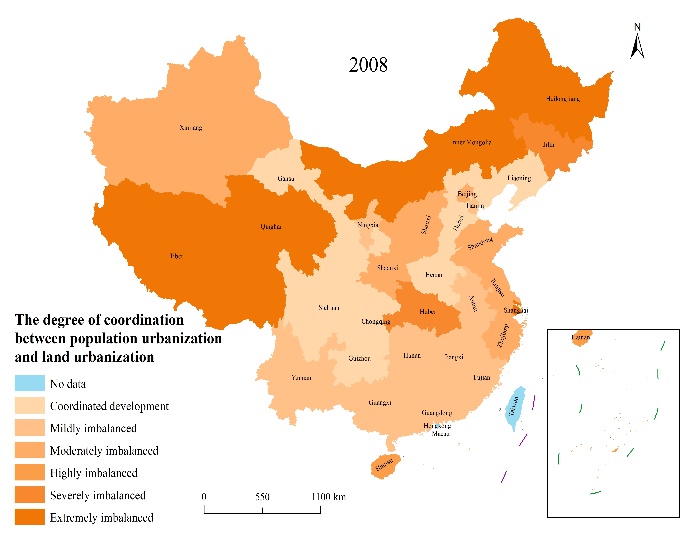

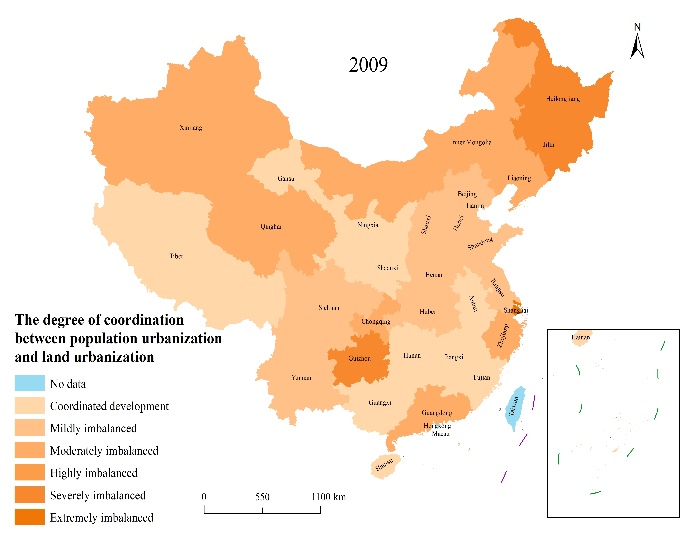

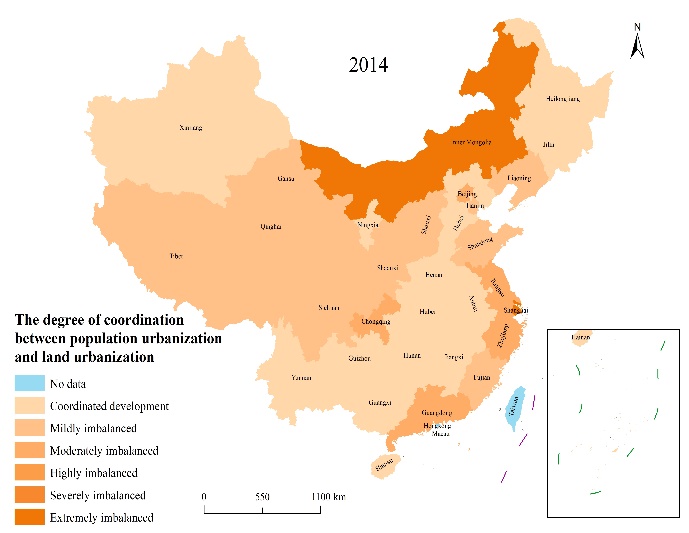

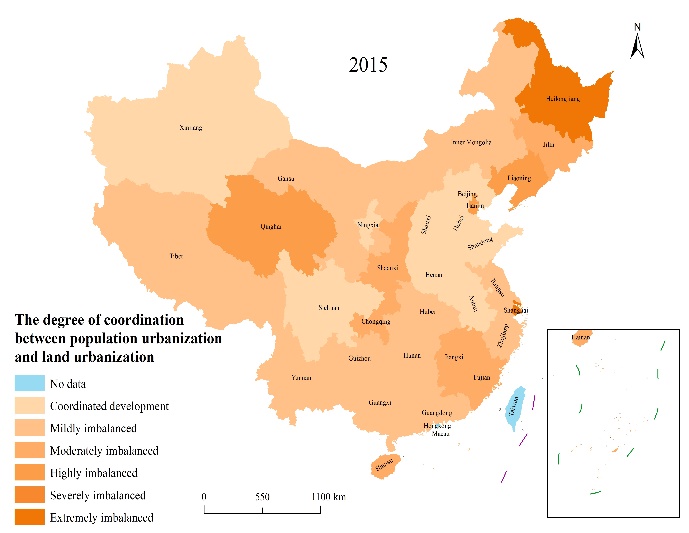

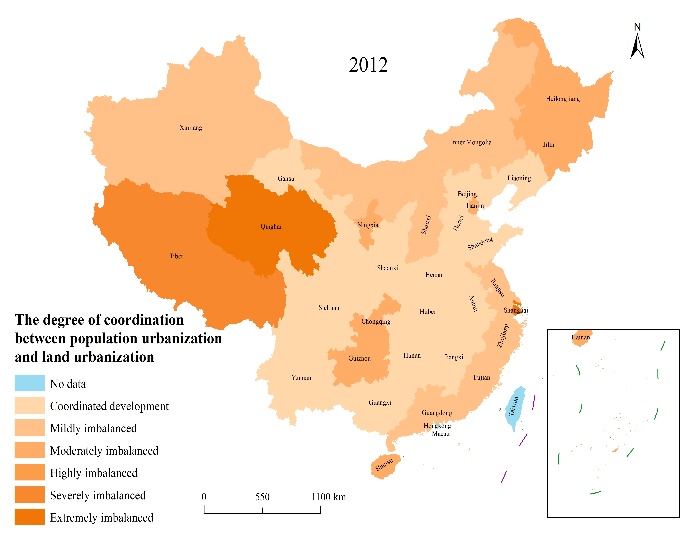

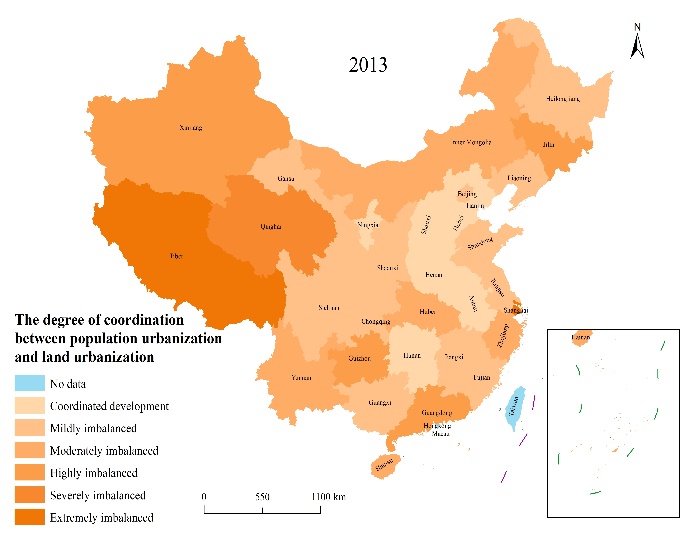

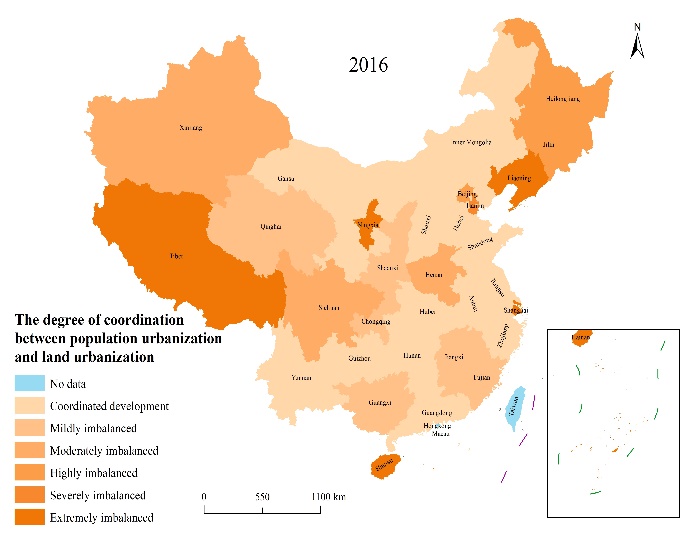

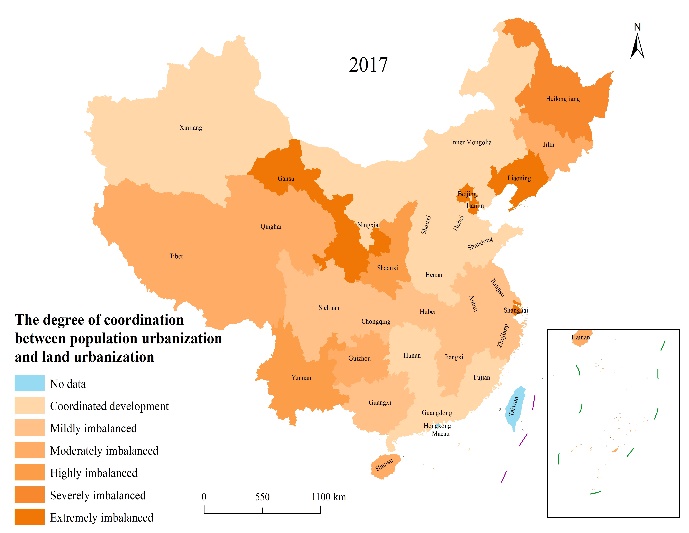

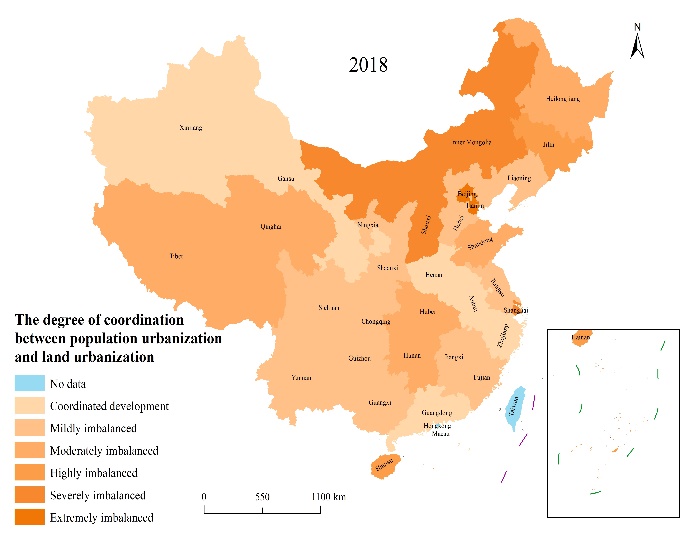

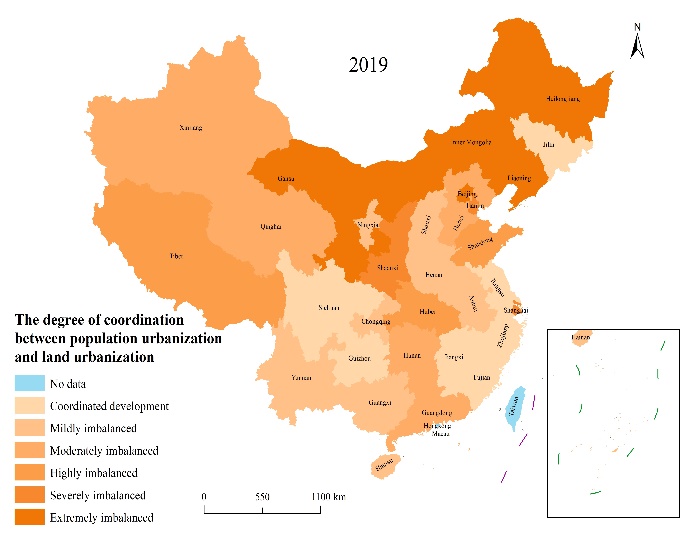


**
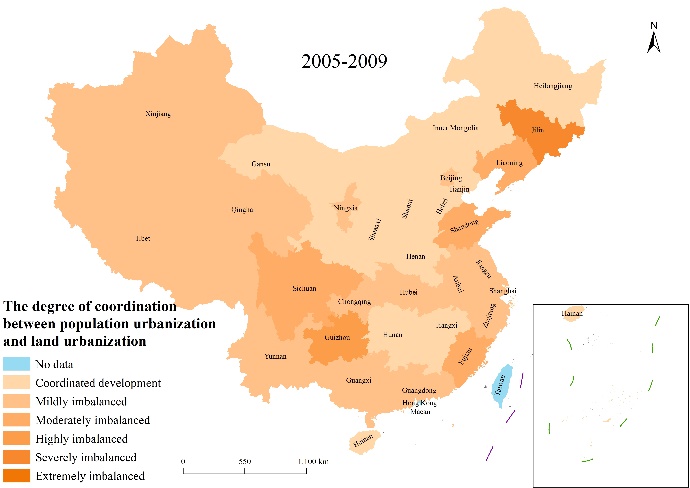

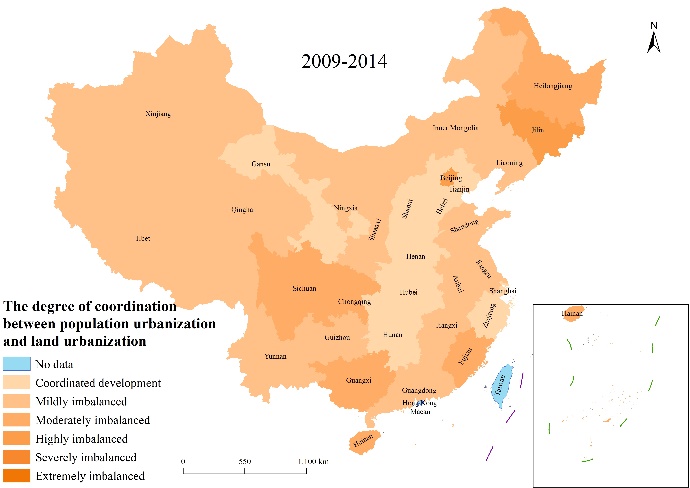
**

**
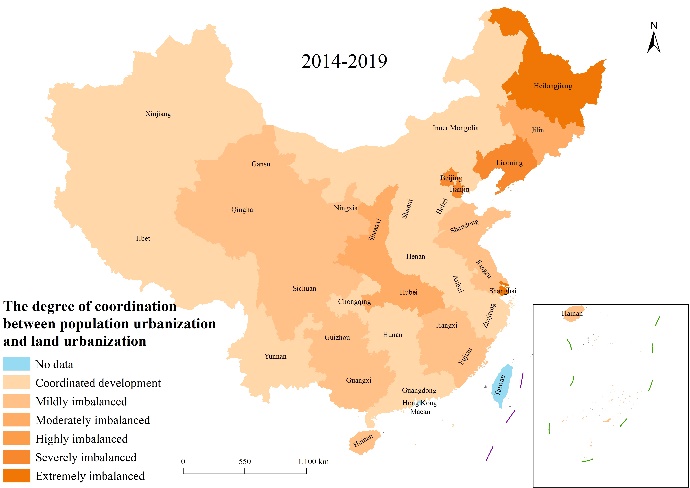
**
